# Increased detection of *Plasmodium knowlesi* in Sandakan division, Sabah as revealed by PlasmoNex™

**DOI:** 10.1186/1475-2875-12-264

**Published:** 2013-07-31

**Authors:** Xiang Ting Goh, Yvonne AL Lim, Indra Vythilingam, Ching Hoong Chew, Ping Chin Lee, Romano Ngui, Tian Chye Tan, Nan Jiun Yap, Veeranoot Nissapatorn, Kek Heng Chua

**Affiliations:** 1 Department of Parasitology, Faculty of Medicine, University of Malaya, Kuala Lumpur, Malaysia; 2 Department of Molecular Medicine, Faculty of Medicine, University of Malaya, Kuala Lumpur, Malaysia; 3 School of Science and Technology, Universiti Malaysia Sabah, Kota Kinabalu, Sabah, Malaysia

## Abstract

**Background:**

*Plasmodium knowlesi* is a simian malaria parasite that is widespread in humans in Malaysian Borneo. However, little is known about the incidence and distribution of this parasite in the Sandakan division, Malaysian Borneo. Therefore, the aim of the present epidemiological study was to investigate the incidence and distribution of *P. knowlesi* as well as other *Plasmodium* species in this division based on a most recent developed hexaplex PCR system (PlasmoNex™).

**Methods:**

A total of 189 whole blood samples were collected from Telupid Health Clinic, Sabah, Malaysia, from 2008 to 2011. All patients who participated in the study were microscopically malaria positive before recruitment. Complete demographic details and haematological profiles were obtained from 85 patients (13 females and 72 males). Identification of *Plasmodium* species was conducted using PlasmoNex™ targeting the 18S ssu rRNA gene.

**Results:**

A total of 178 samples were positive for *Plasmodium* species by using PlasmoNex™. *Plasmodium falciparum* was identified in 68 samples (38.2%) followed by 64 cases (36.0%) of *Plasmodium vivax*, 42 (23.6%) cases of *P. knowlesi*, two (1.1%) cases of *Plasmodium malariae* and two (1.1%) mixed-species infections (i e, *P. vivax/P. falciparum*). Thirty-five PlasmoNex™ positive *P. knowlesi* samples were misdiagnosed as *P. malariae* by microscopy. *Plasmodium knowlesi* was detected in all four districts of Sandakan division with the highest incidence in the Kinabatangan district. Thrombocytopaenia and anaemia showed to be the most frequent malaria-associated haematological complications in this study.

**Conclusions:**

The discovery of *P. knowlesi* in Sandakan division showed that prospective studies on the epidemiological risk factors and transmission dynamics of *P. knowlesi* in these areas are crucial in order to develop strategies for effective malaria control. The availability of advanced diagnostic tool PlasmoNex™ enhanced the accuracy and accelerated the speed in the diagnosis of malaria.

## Background

Malaria, a tropical disease caused by infection with single-celled parasites of the genus *Plasmodium,* is one of the most deadly parasitic diseases in the world. According to the World Malaria Report 2011, an estimated 3.3 billion people were at risk of malaria in 2010. Of this total, 2.1 billion people were at low risk whereas 1.2 billion people were at high risk (>one case per 1,000 population) and living mostly in the WHO African (47%) and Southeast Asian regions (37%) [1]. Malaria is the most common vector-borne parasitic disease in Malaysia, responsible for 20–40 deaths per year over the last decade, and it is estimated that 3% of total Malaysian population live in malaria risk areas. Around 80% of nationwide cases are found in Malaysian Borneo with 58% occurring in the northern state of Sabah and less than 20% of total malaria cases occurring in Peninsular Malaysia [2].

In Sabah, malaria is transmitted by various *Anopheles* mosquitoes, such as *Anopheles balabacensis*, *Anopheles sundaicus* and *Anopheles flavirostris*[3]. Traditionally, human malaria can be caused by four *Plasmodium* species, which include *Plasmodium falciparum*, *Plasmodium vivax, Plasmodium malariae* and *Plasmodium ovale*. These four *Plasmodium* species are well-recognized to cause human malaria worldwide [4]. Infection caused by *P. falciparum* is severe and may be fatal in the absence of prompt recognition of the disease and its complications, while the disease caused by *P. vivax*, *P. ovale* and *P. malariae* is usually milder and rarely fatal.

Recently, a simian malaria parasite, *Plasmodium knowlesi,* has been recognized as the fifth human malaria species by WHO [5]. *Plasmodium knowlesi* is a malaria parasite of Old World monkeys, which was formerly known to cause malaria only in macaques [6]. However, this parasitic infection is widespread in humans in Malaysia and has been reported from Malaysian Borneo [7-11] and Peninsular Malaysia [8,12-14]. This infection has also been reported in Singapore [15-17], Thailand [18,19], Myanmar [20,21], Philippines [22], Indonesia [23,24], Vietnam [25] and Cambodia [26], pinpointing that the incidence of *P. knowlesi* was generally high in Southeast Asian countries especially Malaysia. Unfortunately, this parasite is life-threatening and has caused mortality in humans [27,28].

The diagnosis of *P. knowlesi* infection can be challenging for an inexperienced microscopist because the early trophozoite stages of this parasite are morphologically similar to those of *P. falciparum*[7,29] whilst the later erythrocytic stages (i.e., late trophozoites, schizonts and gametocytes) of *P. knowlesi* are indistinguishable from *P. malariae*[7], including the presence of the typical band-forms. Due to these morphological similarities between *P. knowlesi* and *P. malariae*, naturally acquired *P. knowlesi* infections are often misdiagnosed via microscopy as *P. malariae*[7,29].

This was evident when six out of 14 malaria deaths reported in Sabah during 2010–2011 [28] were actually caused by *P. knowlesi* (later diagnosis done with molecular tools) but were diagnosed initially as *P. malariae* infections via microscopy detection. Hence, in order to overcome the limitations of microscopy for the detection of malaria, molecular tools such as polymerase chain reaction (PCR) are necessary for accurate identification of *P. knowlesi* as well as other *Plasmodium* parasites in order to avoid mortality. This method has proven to be more specific and sensitive than conventional microscopy [30]. Recognizing this advantage, the utilization of molecular techniques in diagnosis and research in molecular epidemiology has become more common. Recently, in a study based on nested PCR assay, a total of 107 malaria cases (44% of 243 samples) have been reported in four districts in the interior division of western Sabah (i.e., Keningau, Tenom, Tambunan, Nabawan) [31].

However, information on malaria incidence in eastern districts of Sabah is scarce. Therefore, the aim of the present epidemiological study was to investigate the incidence and distribution of the *Plasmodium* species in these districts of Sabah, Malaysian Borneo based on a most recent developed hexaplex PCR system in order to obtain accurate epidemiological data which serves as an important foundation in the effectiveness of malaria control strategy. The straightforward single-step hexaplex PCR system (commercial name PlasmoNex*™*) targeting five human *Plasmodium* 18 s small subunit rRNA (ssu rRNA) was developed by Chew *et al.*[32]. This system can detect all five human *Plasmodium* species simultaneously as well as mixed infections in a single step PCR with high sensitivity and specificity. Therefore, this system was the method of choice for the detection of malaria parasites in this study instead of nested PCR because it is rapid and less labour-intensive.

## Methods

### Study area and population

Sabah is one of the 13 member states of Malaysia. It is situated at the northern part of Borneo Island and it shares a land border with Sarawak and Indonesia. This state covers an area of 73,620.1 sq km, comprising of 24 districts with a population of 3,120,040 people. Sabah is a famous tourist destination (e.g., Mount Kinabalu and Sipadan Island). Currently, there are more than 30 officially recognized ethnic groups in Sabah, the largest indigenous groups being the Kadazan-Dusun people, followed by Bajau and Murut. Chinese is the largest non-indigenous ethnic in Sabah. In this research, samples of the studied subjects were obtained from four districts (i.e., Kinabatangan, Beluran, Sandakan, Tongod) within Sandakan division which is the largest division in Sabah. It has approximately 19.4% of Sabah's total population and covers an area of 28,205 sq km. A majority of the ethnic groups in Sandakan division are Chinese, Suluk and Bajau Simunul. Tongod is at the central part of Sabah, whereas Kinabatangan, Beluran and Sandakan are situated in the eastern part of Sabah.

The major economic contributions for Sandakan division are oil palm plantation, timber and eco-tourism. Most of the indigenous populations in this region reside in communities close to palm oil plantations or dense rain forest. This region is surrounded by forest fringe, contains an array of wildlife habitat, which usually act as a natural habitat for the hosts of *P. knowlesi,* the long-tailed (*Macaca fascicularis)* and pig-tailed (*Macaca nemestrina)* macaques as well as the vectors (i.e., *Anopheles leucosphyrus* group of mosquitoes).

### Clinical sample collection

A total of 189 whole blood samples were collected from Telupid Health Clinic, Sabah, Malaysia, from 2008 to 2011. Telupid Health Clinic is the only diagnostic laboratory in Sandakan Division outside of Sandakan town. Usually, patients in Sandakan town receive treatment at the Sandakan Hospital. However, this hospital is about 130 km away from Telupid Health Clinic and it takes about 2–3 hours drive to get there. Therefore, samples from Sandakan and neighbouring districts may be sent to Telupid Health Clinic for diagnosis. Blood samples were collected from Blood Film for Malaria Parasites (BFMP)-positive patients who attended the clinic. The patients who participated in the study were all microscopically malaria positive patients before recruitment. Blood samples included in the study were collected from Sandakan and neighbouring districts such as Kinabatangan, Beluran and Tongod. In this study, 3 ml of blood was collected from each BFMP-positive patient that attended the clinic by qualified medical laboratory staff. A small amount of each sample was used for routine haematological profiling in the laboratory, and genomic DNA was extracted from the remaining sample. Basic demographic data of the patients if available, was obtained and recorded. Complete demographic details and haematological profiles were obtained from 85 patients (13 females and 72 males).

### Ethics statement

Patients’ consent was obtained prior to blood sample collection. For all child participants, parents/guardians provided the consent on their behalf. This study was approved by the Ethics Committee reviewer board of University of Malaya Medical Center (UMMC), Malaysia (MEC reference number 709.2).

### Laboratory procedures

#### ***Conventional microscopic examination***

Blood smears were prepared prior to anti-malarial treatment for patients with suspected malaria. The thick and thin blood smears were stained with Giemsa and examined by experienced microscopist at the Telupid Health Clinic, Sabah. All blood smears were examined under the microscope at a magnification of ×1000 with immersion oil. Routine diagnosis reports were prepared by medical laboratory staff at this clinic. Species of parasites were determined based on the morphology of parasites. Parasite developmental stages such as early trophozoite, late and mature trophozoite, schizonts, and gametocytes were determined and recorded.

#### ***Determination of parasitaemia***

The parasitaemia of infected patients in this study was estimated using a simple modified method, which involved counting of parasites in thin blood films by using the plus system [33]. A code of between one to four plus signs was used to estimate the parasitaemia of parasites whereby + = 1–10 parasites per 100 thin film fields or ~4-40 parasites/μL, ++ = 11–100 parasites per 100 thin film fields or ~ 41–400 parasites/μL, +++ = 1–10 parasites per single thin film fields or ~401-4,000 parasites/μL, and ++++ = more than ten parasites per single thin film fields or >4,000 parasites/μL of blood [11]. Blood films were interpreted as negative if no parasite was observed in 200 microscopic fields.

#### ***Haematological profiles***

Haematological profiles of patients such as haemoglobin level (Hb), platelet counts and total white blood cell counts (TWBC) were obtained and analysed. All these laboratory procedures were carried out at the Telupid Health Clinic, Sabah. Anaemia was confirmed when the Hb level is <110 g/L for children aged six months to two years, <115 g/L for children five to 11 years, <120 g/L for children 12–14 years, <120 g/L for women and <130 g/L for men [34]. Thrombocytopaenia was determined when platelet counts were <150,000 cells/cmm. Patients were divided into three subgroups based on the platelet count. Thrombocytopaenia was considered severe if <50,000 cells/cmm, moderate if 50,000-100,000 cells/cmm and mild if 100,000-150,000 cells/cmm. TWBC count for a normal adult is 4.3-10.8 cells/L and children have a slightly higher TWBC count which is 4.5-11.0 cells/L. Leukopaenia was determined when the TWBC count for an adult was 3.0-5.0 cells/L, and for leukocytosis was 11.0-17.0 cells/L.

#### ***Extraction of genomic DNA***

DNA template for the hexaplex PCR assay was extracted from 200 μl thawed EDTA-blood using the QiAamp DNA Blood Mini Kit (Hilden, Germany), following the manufacturer’s recommendations. Approximately 100 μl of DNA template were obtained from the blood and 1.5 μl of DNA template was used for the hexaplex PCR assay. The extracted DNA was kept in -20°C until further use.

#### ***Detection of Plasmodium species using PlasmoNex***™

Identification of *Plasmodium* species was conducted using PlasmoNex™ targeting the 18S ssu rRNA gene [32]. PCR amplification was performed in 15 μL reaction mixture containing 1X PCR buffer, 3.0 mM MgCl_2_, 0.2 mM of dNTP mix, *Plasmodium* primer mixture, 1 U of *Taq* DNA polymerase and 1.5 μL (~10 ng) of template DNA. Cycling condition of the PCR amplification was as follows: initial denaturation at 95°C for 5 min, 35 amplification cycles at 95°C for 30 sec, 56°C for 30 sec, and 65°C for 40 sec, followed by final extension at 65°C for 10 min in a thermal cycler (BIO-RAD, Germany). The amplified products were visualized on a 3.0% (w/v) agarose gel stained with ethidium bromide.

### Statistical analysis

Statistical analysis was carried out using the SPSS software programme for Windows version 17 (SPSS, Chicago, IL, USA). The intensity of malaria infections (parasitaemia) was estimated by a simple modified method which involved counting of parasite numbers in thin blood films by using the plus system and was categorized into four main categories: +, ++, +++ and ++++. For descriptive data, percentage was used to describe the characteristics of the studied population, including the prevalence of anaemia, thrombocytopaenia, leukopaenia and leukocytosis. The distribution of Hb, platelet and TWBC were presented as median and interquartile range (IQR) after being examined for normality using the Shapiro-Wilk test. The correlation between continuous variables was examined using Spearman’s correlation coefficients (r_s_) test as they were not normally distributed. A Pearson’s Chi-square (X^2^) test was used to test the association between each variable. The level of statistical significance was set at p < 0.05 for each test. For each of the statistically significant variable, odd ratios (ORs) and 95% confidence interval (95% CI) were calculated to explore the association between variables of interest.

## Results

### Incidence and distribution of *Plasmodium* species based on microscopy

A total of 189 BFMP-positive samples were collected during the study period at Telupid Health Clinic, Sabah. Based on microscopy, 38.6% (73/189) were *P. falciparum*, 33.9% (64) were *P. vivax*, 25.9% (49) were *P. malariae*, 1.1% (two) were mixed *P. vivax/P. malariae* and 0.5% (one) was mixed *P. falciparum/P. vivax* infections (Table 1). Of the *P. vivax*-positive blood films, 92.2% (59/64) had asexual parasites only, 7.8% (five) had both asexual parasites and gametocytes. For *P. falciparum-*positive blood films, 60.3% (44/73) had asexual parasites alone, 39.7% (29) had asexual parasites and gametocytes. As for *P. malariae*-positive blood films, 98.0% (48/49) had asexual parasites alone while only 2.0% (1/49) had both asexual parasites and gametocytes (Table 2). There was no *P. knowlesi* and *P. ovale* malaria infection reported from the microscopic examination.

**Table 1:** **Comparison of diagnosis of *Plasmodium *species by microscopy and PlasmoNex™ for the samples collected from eastern and central Sabah (N = 189)**

	**No. of cases identified based on PCR examination**						
**Microscopy results**	***P. falciparum***	***P. vivax***	***P. knowlesi***	***P. malariae***	***P. falciparum and P. vivax***	**Negative**	**No. of cases identified by microscopy**
***P. falciparum***	62	2	4	0	0	5	73
***P. vivax***	0	58	3	0	0	3	64
***P. malariae***	6	3	35	2	0	3	49
***P.vivax *and *P. malariae***	0	1	0	0	1	0	2
***P. falciparum *and *P. vivax***	0	0	0	0	1	0	1
**Total**	68	64	42	2	2	11	189

**Table 2:** **Parasite developmental stage in this study (n = 186)**^**a**^

**Species**	**Developmental stages**				
	**N**	**Asexual**		**Asexual + Gametocytes**	
		**n**	**%**	**n**	**%**
***P. falciparum***	73	44	60.3	29	39.7
***P. vivax***	64	59	92.2	5	7.8
***P. malariae***	49	48	98.0	1	2.0
*P. knowlesi	42	40	95.2	2	4.8

^**a**^Mixed infections were not included.

*PlasmoNex™ confirmed *P. knowlesi* cases.

Given that the complete demographic details and haematological profiles could only be obtained from 85 patients (i.e., 13 females and 72 males), the results pertaining to demographic and haematological profiles will be based on 85 and not 189 patients. The ethnicities of patients that participated in this study were Dusun (50.6%), Bugis (18.8%), Filipinos (5.9%), Rung (4.7%) and others . Among the four districts of Sandakan division, Kinabatangan has the highest malaria cases detected (35 cases; 41.2% of 85) in this study followed by Sandakan (22 cases; 25.9%), Beluran (16 cases; 18.8%) and lastly Tongod (12 cases; 14.1%).

With regard to the age groups, there was a total of 19 children and 66 adults. The age range was between two to 60 years old (mean = 24.8 years; median = 24.0 years). Malaria was more prevalent among adults (77.6%) compared to children (22.4%). With regard to gender, male patients (84.7%) were almost five-fold more compared to female patients (15.3%) (Table 3). Generally, malaria was prevalent among adults especially male.

**Table 3:** **Distribution of hematological status among malaria infected patients in this study (n = 85)**^**a**^

		**Hb (g/l)**	**Platelet (cells/cmm)**	**TWBC (10**^**9**^**cells/L)**	**Anaemia**^**b**^		**Thrombocyto-penia**^**c**^		**White blood cell count**			
									**Leukopenia**^**d**^		**Leukocytosis**^**e**^	
**Age/Gender**	**N**	**Median (IQR)**	**Median (IQR)**	**Median (IQR)**	**No.**	**%**	**No.**	**%**	**No.**	**%**	**No.**	**%**
**Age (year)**												
Children (0–14)	19	107(86–111)	102000 (73000–180000)	5.9 (5.3-9.3)	14	73.7**﹡**	9	47.4	2	10.5	1	5.3
Adult (>15 years)	66	134 (121–142)	88500 (55750–127000)	6.75 (5.0-8.6)	27	40.9	39	59.1	5	7.5	5	7.6
**Gender**												
Male	72	132 (119–142)	94000 (61500–132750)	6.6 (5.2-8.3)	31	43.1	40	55.6	5	6.9	6	8.3
Female	13	107(75–114)	87000 (60500–131500)	6.9 (4.4-9.6)	10	76.9**﹡**	8	61.5	2	15.4	0	0
*Total	**85**	**128 (109–140)**	**91000 (61500–132500)**	**6.6 (5.1-8.8)**	**41**	**48.2**	**48**	**56.5**	**7**	**8.2**	**6**	**7.1**

*Total samples were based on 85 microscopic positive samples that had both complete demographic and hematological data.

N: number examined; IQR: inter quartile range.

^a^Distribution of anaemia, thrombocytopenia, leukopenia and leukocytosis according to gender and age groups was based on 85 microscopic positive samples.

^b^Haemoglobin (Hb) below age-specific threshold (see methodology).

^c^Platelet counts <150,000 cells/cmm.

^d^Total white blood cells counts was 3.0-5.0 cells/L.

^e^Total white blood cells counts was 11.0-17.0 cells/L.

﹡Significant difference (p < 0.05).

According to the haematological profiles, the present study showed that the overall median concentration of Hb, platelet count and TWBC for the patients was 128.0 g/l (IQR = 109.0-140.0), 91,000 cells/cmm (IQR = 61,500-132,500) and TWBC was 6.6 × 10^9^ cells/L (IQR = 5.1-8.8), respectively (Table 3). Overall, the Hb level among malaria patients were at the borderline of normal Hb level (>120 g/l for woman and >130 g/l for man), however, the platelet level of patients were much lower than the normal platelet count (>150,000 cells/cmm). TWBC of malaria patients in this study were within the normal range (4.5-10.0 × 10^9^ cells/L). The median Hb value (107 g/l; range: 86–111) and TWBC count (5.9 × 10^9^ cells/L; range: 5.3-9.3) among infected children were lower than the Hb value (134 g/l; range: 121–142) and TWBC count (6.75 × 10^9^ cells/L; range: 5.0-8.6) of adults. However, the platelet count among infected children (102,000 cells/cmm; range: 73,000-180,000) was higher when compared to the adults (88,500 cells/cmm; range: 55,750-127,000). With regard to gender, male had higher Hb value (132 g/l; range: 119–142) and platelet count (94,000 cells/cmm; range: 61,500-132,750) compared to female’s Hb value (107 g/l; range: 75–114) and platelet count (87,000 cells/cmm; range: 60,500-131,500). However, the TWBC count of female (6.9 × 10^9^ cells/L; range: 4.4-9.6) was slightly higher than male (6.6 × 10^9^ cells/L; range: 5.2-8.3).

Study showed that an overall of 48.2% (41/85) of the patients had anaemia, 56.5% (48/85) had thrombocytopaenia, 8.2% (7/85) had leukopenia and 7.1% (6/85) had leukocytosis (Table 3). The prevalence of anaemia, thrombocytopaenia, leukopaenia and leukocytosis were further analysed according to age and gender (Table 3). The prevalence of anaemia was found to be significantly associated with age and gender. The prevalence of anaemia was significantly higher in children (x^2^ = 6.30; p = 0.012) compared to adult. Study also showed that children had higher risk (OR = 1.8; 95% CI = 1.213-2.674) of getting anaemia compared to the adults. With regard to gender, it was observed that anaemia was significantly higher in female (x^2^ = 5.1; p = 0.025) compared to male. The risk of female suffering anaemia was 1.8 times higher than male (OR = 1.8; 95% CI = 1.199-2.663).

However, the prevalence of thrombocytopaenia, leukopaenia and leukocytosis were not significantly associated with age and gender. Although the prevalence of thrombocytopaenia in adult (59.1%) was higher compared to children (47.4%), the difference was not statistically significant. Similarly, there was no significant difference between leukopaenia and leukocytosis with age although the prevalence of leukopaenia was higher in children (10.5%) and leukocytosis was higher in adult (7.6%). There was also no significant difference between thrombocytopaenia, leukopaenia and leukocytosis with gender.

### Incidence and distribution of *Plasmodium* species based on PlasmoNex™

Out of the 189 samples analysed, a total of 178 samples were positive for *Plasmodium* species by using PlasmoNex™, however, 11 (5.8%) samples were found to be negative. *Plasmodium falciparum* was identified in 68 samples (38.2%) which was the highest number of species detected by PlasmoNex™ in this study followed by 64 cases (36.0%) of *P. vivax* and 42 (23.6%) cases of *P. knowlesi* and two (1.1%) cases of *P. malariae*. Overall, only two (1.1%) of the samples were found to have mixed-species infections (i.e., *P. vivax/P. falciparum*) by PlasmoNex™. For PlasmoNex™ confirmed *P. knowlesi* infection, there were only 4.8% of the samples (2/42) that had both asexual parasites and gametocytes while 95.2% of the samples (40/42) had only asexual stages.

**Figure 1:**
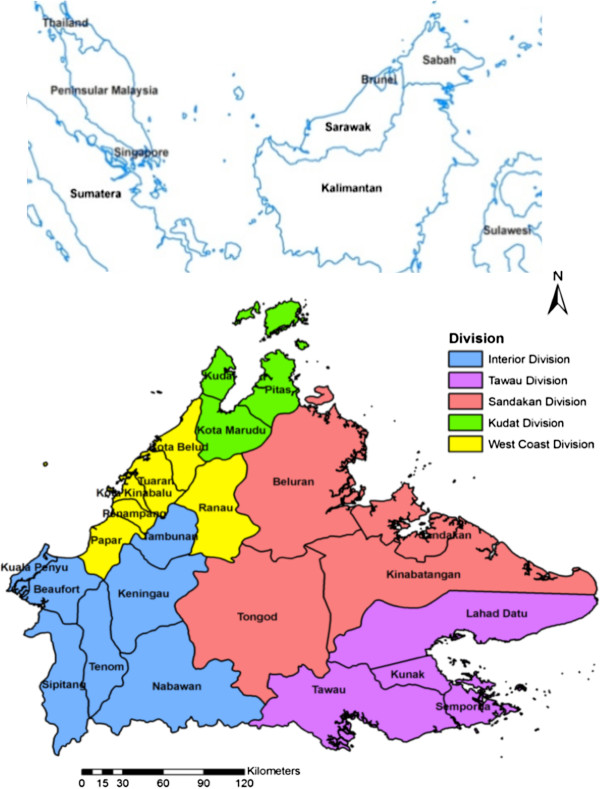
**Location of human *Plasmodium knowlesi *infection in Sabah, Malaysia.** The inset map of Southeast Asia shows the location of Sabah. Note: The location of human *P. knowlesi* detected in this study is marked by triangles. Stars represented the distribution of this parasite reported by previous studies [10,35-38].

Of the 49 samples which were reported as *P. malariae* by microscopy examination, only two (1.1%) were found to be *P. malariae* by PlasmoNex™ (Table 1). Thirty-five (71.4% of 49) cases of *P. knowlesi* were microscopically misdiagnosed as *P. malariae* in this study. Besides, four cases of *P. knowlesi* were microscopically misdiagnosed as *P. falciparum* and three cases of *P. knowlesi* were also misdiagnosed as *P. vivax* (Table 1). Interestingly, *P. knowlesi* was detected in all four districts in Sandakan division and Kinabatangan had the highest incidence of *P. knowlesi* (31.6%). Figure 1 showed the location of *P. knowlesi* cases detected in this study. No *P. ovale* was detected by using PlasmoNex™. Six *P. falciparum* cases were misdiagnosed as *P. malariae* by microscopy examination as well as one mixed infection of *P. falciparum*/*P. vivax* was misdiagnosed as *P. vivax*/*P. malariae* (Table 1).

Of the 178 PlasmoNex™ positive samples, basic demographic data was obtained from 70 patients (69 with single infection, one with mixed infections). The haematological analysis of the present study focused only on single infection. Data showed that the patients within the age group 21–30 years (39.1%) were the most susceptible group to malaria in this study (Table 4). There was a greater proportion of adult patients with *P. vivax* (24.6%) compared to children (8.3%) whereas the infection percentage for both *P. falciparum* and *P. knowlesi* patients was almost the same for adult and children. The oldest age range for patients with *P. knowlesi* infection were slightly higher (51–60 years old) than that of patients with *P. falciparum* and *P. vivax* (41–50 years old). The youngest child with PCR-confirmed *knowlesi* malaria was nine years old. This showed that patients with *P. knowlesi* infection demonstrated a wide age distribution (i.e., nine to 60 years). Among *P. knowlesi* infected patients, the percentage of male patients (95.0%) was much higher compared to female patients (5.0%) (Table 4).

**Table 4:** Demographic data for PCR-confirmed malaria positive patients in this study (n = 69)

**Characteristics**			***P. falciparum***		***P. vivax***		***P. knowlesi***		***P. malariae***	
	**N**	**%**	**n**	**%**	**n**	**%**	**n**	**%**	**n**	**%**
**Gender**										
Male	58	84.1	27	46.6	11	19.0	19	32.8	1	100.0
Female	11	15.9	6	54.5	4	36.4	1	9.1	0	0
X^2^				0.2		1.6		2.5		0.2
P				0.627		0.200		0.113		0.661
**Age groups**										
Children (0–14 years)	12	17.4	6	50.0	1	8.3	4	33.3	1	8.3
Adults (>15 years )	57	82.6	27	47.4	14	24.6	16	28.1	0	0
X^2^				0.03		1.8		0.1		4.8
P				0.868		0.177		0.715		0.028
**Age groups**										
0-10	8	11.6	4	28.6	1	7.1	2	14.3	1	7.1
11-20	14	20.3	6	37.5	4	25.0	4	25.0	0	0
21-30	27	39.1	13	41.9	7	22.6	7	22.6	0	0
31-40	11	15.9	6	50.0	1	8.3	4	33.3	0	0
41-50	6	8.7	4	50.0	2	25.0	0	0	0	0
51-60	3	4.3	0	0	0	0	3	75	0	0
X^2^				4.4		4.0		0.7		5.1
P				0.500		0.542		0.084		0.400
*Total	**69**	**100.0**	**33**	**47.8**	**15**	**21.7**	**20**	**29.0**	**1**	**1.5**

*Total samples were based on 69 PCR positive samples. Mixed infection was not included.

Spearman’s correlation co-efficients (r_s_) test was used to study the relationship between Hb value, platelet count and white blood cell count with parasite density. Overall, there was a significant correlation between parasite density with Hb value and platelet value but not with white blood cell count for all the species. There was a significant inverse correlation between parasite density with Hb value and platelet value. Moderate inverse correlation was noted between increasing parasite density and decreasing Hb value (r_s_ = -0.317; p = 0.003). Likewise, the parasite density also significantly correlated with platelet value. Increasing parasite density correlated with decreasing platelet value (r_s_ = -0.262, p = 0.015) for all the *Plasmodium* species. As for TWBC, no significant correlation was observed with parasite density.

Anaemia was common in all species, occurring in 13 (39.4%), nine (60.0%) and nine (45.0%) patients with *P. falciparum*, *P. vivax* and *P. knowlesi* malaria respectively (Table 5). Among patients with malaria parasite density ‘++++’, *knowlesi* malaria patients showed the highest percentage of anaemia (80.0%). Besides, the overall median concentration of platelet count for *knowlesi* patients (75,500 cells/cmm) was the lowest platelet counts compared to *P. falciparum* (94 000 cell/cmm) and *P. vivax* (103,000 cells/cmm) malaria patients (Table 5). The TWBC level was almost the same regardless of malaria species.

**Table 5:** **Intensity of malaria infections and the distribution of hematological status among malaria patients in this study (n = 69)**^**a**^

**Malaria species and **^**b**^**parasitemia**		**Hb (g/I)**	**Platelet (per/μl)**	**TWBC (10**^**3 **^**cells/μl)**	**Anaemia**	
	**N**	**Median**	**Median**	**Median**	**No.**	**%**
***P. falciparum***						
+	5	140	127000	6.3	2	40.0
++	4	141	75500	7.0	0	0
+++	8	129	92500	6.3	4	50.0
++++	16	120.5	75500	8.2	7	43.8
**Total**	33	132	94000	6.8	13	39.4
***P. vivax***						
+	1	120	301000	10.3	1	100.0
++	6	135	108000	5.2	3	50.0
+++	4	120	127500	7.4	2	50.0
++++	4	118.5	71500	6.5	3	75.0
**Total**	15	121	103000	6.3	9	60.0
***P. knowlesi***						
+	1	130	63000	5.3	1	100.0
++	5	140	82000	7.8	1	20.0
+++	9	132	77000	6.7	3	33.3
++++	5	120	73000	6.6	4	80.0
**Total**	20	130.5	75500	6.7	9	45.0
*Overall	**69**	130	89000	6.6	31	44.9

*Mixed infection was not included.

^a^Total samples were based on 69 PCR positive samples with complete hematological profiles.

N: number examined.

^b^Parasitemia:

+ = 1–10 parasites per 100 thin film fields.

++ = 11–100 parasites per 100 thin film fields.

+++ = 1–10 parasites per single thin film fields.

++++ = more than 10 parasites per single thin film fields.

The platelet count was further analysed according to malaria species. Thrombocytopaenia (platelet count <150,000 cells/cmm) was observed in all malaria species. There were 13 patients who had severe thrombocytopaenia, 27 patients had moderate thrombocytopaenia and 15 had mild thrombocytopaenia whereas 14 patients had no thrombocytopaenia (Table 6). Data showed that malaria patients were more frequently found to have moderate thrombocytopaenia (39.1%; 27/69) in this study. Patients with *falciparum* malaria (6/13; 46.2%) were found to have the highest cases of severe thrombocytopaenia followed by *knowlesi* malaria patients (5/13; 38.5%) and *vivax* malaria patients (1/13; 7.7%). This showed that *P. falciparum* and *P*. *knowlesi* patients had higher risk of getting severe thrombocytopaenia compared to *P. vivax* patients*. Plasmodium falciparum* patients also had the highest number of moderate thrombocytopaenia (12/27; 44.4%) followed by *P. knowlesi* infected patients (9/27; 33.3%) and six of them were *P. vivax*-infected patients (6/27; 22.2%) (Table 6).

**Table 6:** Platelet counts in malaria patients according to different species detected by PCR in this study (n = 69)

	**Human Plasmodium species**								
**Laboratory results**	**N**	***P. falciparum***		***P. knowlesi***		***P. vivax***		***P. malariae***	
		**No.**	**%**	**No.**	**%**	**No.**	**%**	**No.**	**%**
**Thrombocytopenia**									
Severe thrombocytopenia <50,000 cells/cmm	13	6	46.2	5	38.5	1	7.7	1	7.7
Moderate thrombocytopenia 50,000-100,000 cells/cmm	27	12	44.4	9	33.3	6	22.2	0	0
Mild thrombocytopenia 100,000-150,000 cells/cmm	15	7	46.7	3	20.0	5	33.3	0	0
No thrombocytopenia >150,000 cells/cmm	14	8	57.1	3	21.4	3	21.4	0	0
*Total	**69**	**33**	**47.8**	**20**	**29.0**	**15**	**21.7**	**1**	**1.4**

*Mixed infections were not included.

## Discussion

Malaria is one of the most severe parasitic infections and it is also a major public health problem in many countries in Southeast Asia. In the 1980s, the prevalence of malaria drastically decreased and was eliminated in many areas of Peninsular Malaysia due to the efficient malaria control programme. However, malaria is still prevalent in Malaysian Borneo and among the ethnic minority groups [39]. Recently, significant reductions in malaria cases have been observed and achieved in Malaysia [1]. Malaysia is now working to be malaria-free in the peninsula by 2015 and in Malaysian Borneo by 2020 [1,40]. However, recently, a significant increase (>ten-fold) of *P. knowlesi* cases was observed in Sabah between 2004 and 2011 and this trend threatens malaria elimination [41]. Since the early 2000s, it was reported that most districts of Sabah experienced an increase of *P. knowlesi* cases and it appeared to have begun in the south-west of Sabah (Interior division), followed by West Coast division and gradually progressed north-easterly (Kudat division). This increase appears to have begun initially in the Interior division and there was a steady increase of *P. knowlesi* cases from 2000 to 2011 in this region. West Coast division experienced an increase of *P. knowlesi* cases later, from 2001 to 2009. After that, the tip of Borneo, Kudat division experienced the most noticeable increase in *P. knowlesi* cases with more than 200 cases for the years 2009 and 2011. *Plasmodium knowlesi* infection has also been reported in eastern districts of Sabah (Sandakan and Tawau), although the cases reported in these two districts were fewer compared to other districts, it has been increasing since 2008 [41]. Findings of the present study showed that *P. knowlesi* cases were also detected in the districts of Sandakan division. This shows that *P. knowlesi* is generally prevalent in Sabah. Therefore, the main objective of this study is to investigate the incidence and distribution of *P. knowlesi* as well as other human *Plasmodium* species in eastern Sabah.

Hexaplex PCR (or PlasmoNex™) findings of the present study demonstrated that *P. knowlesi* were identified in 42 (23.6%) samples, however, microscopic examination did not show *P. knowlesi* in any samples examined. Thirty-five infections diagnosed as *P. malariae* by microscopic examination were found to be *P. knowlesi*. This finding was similar to the studies carried out by other researchers in Malaysian Borneo [7,10,28,35], reporting that most microscopy-positive *P. malariae* infections were actually proven to be *P. knowlesi* infections when molecular approach was used. However, the transmission dynamics of this infection still remain unknown despite wide distribution of this parasite in Malaysian Borneo. The most prevalent *Plasmodium* species identified in the study was *P. falciparum* followed by *P. vivax* and *P. knowlesi*. It was reported that *P. vivax* was the most prevalent human malaria parasites in Malaysia [42], however, *P. falciparum* was found to be the most prevalent species in this study.

Conventional diagnostic technique, microscopic examination of asexual stages of *Plasmodium* on thin and thick blood films has been considered as the “gold standard” for the diagnosis of *Plasmodium* parasite infection because it is cost-effective and simple [43]. However, it can be rather challenging to diagnose mixed infections and infections with low level parasitaemia [44]. The diagnostic accuracy depends greatly on the experience of microscopists. Sensitivity of microscopy examination of malaria parasites can be affected by various factors such as the quality of staining, skills of the laboratory technician, expertise of microscopists and types of microscope used. In addition, microscopic identification of *P. knowlesi* is rather challenging due to its morphology, which is indistinguishable from *P. falciparum* and *P. malariae*. Underdiagnosis of *P. knowlesi* by microscopy examination was observed in the present study. Thus, molecular tool, such as PCR, is necessary to overcome the limitations of microscopy for malaria detection especially in generating epidemiology data.

The recently developed PlasmoNex™ [32], was used for the detection of malaria parasites and identification of the five human *Plasmodium* species in this study. Molecular methods based on DNA amplification have been used for malaria diagnosis since 1980s [45-49]. Several reports have shown that the DNA-based amplification methods had higher sensitivity (as low as 1 parasite/μl of blood) compared to microscopic examination of thin blood films, especially in cases of low parasitaemia or mixed infections [50-52]. Besides that, they are able to characterize each human malaria species [45,53-55].

PlasmoNex™ is a single-step hexaplex PCR system [32], which is able to detect all five species of human malaria with sensitivity below 0.5 parasites/μl of blood sample. To date, nested PCR is the molecular gold standard for the diagnosis of malaria and species identification. This method has been proven to be more specific and sensitive than conventional microscopy [30]. However, malaria diagnosis and species identification of malaria by nested PCR or semi-nested PCR are time consuming. The time required for hexaplex PCR is only three hours compared to semi-nested PCR which requires about five hours and approximately 14 hours for nested PCR, including the time for PCR preparation and gel electrophoresis. Besides, there are some other advantages of hexaplex PCR over the available nested PCR as it is less labour intensive, less contamination and reduced usage of reagents and consumables. Hexaplex PCR is a single-step system whereas semi-nested PCR required at least two PCR reactions and for nested PCR at least six PCR reactions are needed for the identification of all five *Plasmodium* species. The fewer PCR reactions reduce the usage of reagents and consumables and are also less labour intensive. Mixed infection detected by single-step hexaplex PCR in this study indicated that this system is robust and capable of detecting at least up to two species level mixed infections.

Recently, *P. knowlesi* cases had been reported in the Interior division and north-eastern state of Sabah [10,28,35]. It is obvious that the prevalence of *P. knowlesi* in Sabah has increased markedly and studies reported on the discovery of this parasite by using molecular approaches have greatly increased recently. In the early 1990s, there was around 50,000 malaria cases reported in Sabah and a continuous decrease was observed from the year 2001 to 2003. There were 6,050 cases in 2001, and this number fell to 5,096 cases in 2002 and subsequently to 1,770 cases in 2003 [56]. Previous report also showed that the notification of *P. falciparum* and *P. vivax* in Sabah had drastically decreased over the past decade [41]. The notification of *P. falciparum* and *P. vivax* had decreased to around 600 for the year 2011, however, a significant increase of *P. knowlesi* had occurred following the reduction of *P. falciparum* and *P. vivax* in Sabah. It showed that when human malaria cases decreased, *P. knowlesi* cases were on the rise. It is postulated that environmental changes together with decreasing rates of *P. falciparum* and *P. vivax* were likely to have contributed to the rise of *P. knowlesi* in Sabah [41]. Human activities such as extensive deforestation due to road construction work, oil palm plantation, timber or eco-tourism that occur in Sabah have brought human, macaques and mosquito vector into close contact and this propagates successful transmission of this parasite. In the present study, *P. knowlesi* infection was detected in all four districts of Sandakan divisions with the highest incidence of this parasite in the Kinabatangan region.

The wide distribution of *P. knowlesi* in this region is not surprising as this region is surrounded by dense primary and secondary forests which act as suitable habitats for mosquito vectors (i.e., forest-dwelling *Anopheles balabacensis*) [3] and macaques (i.e., long-tailed macaques, *M. fascicularis* and pig-tailed macaques, *M. nemestrina*) which are the natural hosts for *P. knowlesi* in Sabah. Previously, *An. balabacensis* was well recognized as the predominant vector of human malaria in Sabah [36]. However, *Anopheles donaldi* was reported to have replaced *An. balabacensis* in the Kinabatangan region in 2005 [56]. The peak outdoor feeding time for *An. donaldi* mosquitoes occurs between 18:00 and 19:00. This is the period most people are being bitten by this mosquito when the adults are working outside and children are having outdoor activities. Although *An. balabacensis* was recognized as the vector of human malaria in Sabah, more studies should be carried out to investigate the current vector situation in this region. Perhaps, the situation of vectors in this region has changed due to recent development and mass forest clearance in the area. Nevertheless, communities should be informed to take necessary preventive measures against this parasite in order to prevent outbreaks of *P. knowlesi* infection.

Malaria patients who participated in the study comprised ages from young children to the elderly, however, this infection was more prevalent among adults especially the males. The epidemiological data showed that *P. knowlesi* malaria was diagnosed in male patients more often than in female patients, and only small proportion of *knowlesi* malaria cases occurred in children, this may be related to the limited outdoor or forest activities by this group of patients. These findings were in concordance with those reported from two studies in Sarawak [7,9], where a smaller proportion of *knowlesi* malaria occurred in children and no clustering of cases was reported. Findings of these two studies suggested that *P. knowlesi* infection occurs outside people’s home and adults were the high risk group of this infection due to greater forest exposure, and it is postulated that human to human transmission did not occur. However, previous study postulated that the transmission of *P. knowlesi* may occur from human to human due to the discovery of family clustering cases [57]. Nevertheless, no clustering of *P. knowlesi* cases was found in the present study.

Microscopic findings showed that there were more gametocytes found in the *P. falciparum* and *P. vivax* samples compared to *P. knowlesi* samples*.* This could infer that infected humans were getting the *knowlesi* infection from mosquitoes that fed on monkeys and not from humans. Consistent with previous studies [9,35,57], patients with *P. knowlesi* were older than those with *P. falciparum* or *P. vivax*. The possible reason for the older age group being infected by *P. knowlesi* was discussed in a study done by William and his colleagues [41]. It was suggested that older individuals had greater forest exposure compared to the young. Patients aged between 21 to 30 years old were the group with the highest number of malaria cases detected in this study, possibly due to greater forest activity by this group, who either work or stay in the forest, such as timber and oil palm plantations workers, since the economic activities in Kinabatangan are the timber industry and oil palm plantations and the highest cases of *P. knowlesi* were detected in this district. Long working hours in the forest may increase the risk of exposure to the mosquito vectors, resulting in successful transmission of *P. knowlesi*. Despite these observations, epidemiological risk factor of *P. knowlesi* malaria still remains unclear, hence further investigations are required in order to develop successful control strategies for *knowlesi* malaria.

The symptoms of human *P. knowlesi* infection are almost similar to the other four human malaria species, however, it is more severe than the disease caused by *P. vivax* and more virulent because it has a short life-cycle of 24 hours [58] which leads to hyperparasitaemia enabling a fast progression of the disease. Malaria is frequently associated with a variety of haematological complications such as thrombocytopaenia and anaemia and both have high mortality rates [9,35,57,59-61]. A healthy human platelet count ranges from 150,000 to 450,000 platelets per microlitre of blood, levels lower than this range is considered as thrombocytopaenia. It is one of the common features of acute malaria and this occurs in *P. knowlesi*[9,15,35,62-64], *P. falciparum*[65,66] and *P. vivax*[66] infections regardless of severity of infection.

The present study showed similar results with previous studies whereby thrombocytopaenia occurred in all species of human malaria parasites indicating that thrombocytopaenia is a common feature of malaria and its occurrence should increase the suspicion of malaria. However, there are several other causes of thrombocytopaenia such as leukaemia, hereditary syndromes, HIV-associated thrombocytopaenia and also dengue fever. Thrombocytopaenia can be caused by decreased platelet production or peripheral destruction. It is postulated that malarial antigen caused the lysis of immune complexes, which leads to sequestration of the injured platelet by macrophages in the spleen [67,68]. However, the exact mechanism of thrombocytopaenia in malaria is not clear and requires further investigation. Findings of the present study demonstrated that most of the *P. falciparum* patients suffered severe and moderate thrombocytopaenia followed by *P. knowlesi* and *P. vivax* patients. This might explain the role of platelet activation in the pathogenesis of liver-attacking malaria. Platelets are produced by a process called thrombopoiesis, which occurs in the bone marrow by budding off from megakaryocytes. Thrombopoietin is a hormone that is secreted for the regulation of platelet production and megakaryocytes. This hormone is produced by the liver and kidneys. Hyperparasitaemia of *P. falciparum* and *P. knowlesi* parasites due to their shorter lifecycle may destruct the production of this hormone in the liver and thus decreased the production of platelet and caused thrombocytopaenia. However, this hypothesis requires further investigation. Many studies have shown that *P. knowlesi* patients suffered severe thrombocytopaenia [28,35,69,70]. Findings of the present study were in parallel with the study done by Barber and her colleagues in 2011 [35] where platelet counts of patients were lower in *P. knowlesi* than in *P. falciparum* malaria. Besides, findings of the present study are also in line with the study conducted by Patel and his colleagues [66] which reported that patients with *P. falciparum* malaria were found to have lower platelet counts than patients with *P. vivax* malaria. Therefore, findings of present study demonstrated that the platelet count of *P. knowlesi*-infected patients were lower than those in *P. falciparum*-infected patients and *P. vivax*-infected patients, which, in turn, were lower than those in the uninfected patients and a platelet count below 75,500 cells/cmm should increase the suspicion of *P. knowlesi* infection. Nonetheless, it is suggested that in order to verify this hypothesis, more samples need to be included in future. It is believed that thrombocytopaenia is more common in *P. falciparum* malaria compared to *P. vivax* malaria. However, recent studies have shown the trend that thrombocytopaenia was found to be equally common in *P. vivax* malaria compared to *P. falciparum*[71-74]. A study conducted by George and Alexander [75] reported significant thrombocytopaenia in *P. vivax* malaria. The findings were in accordance with those in Brazil [76] and Qatar [77]. Although the relation between thrombocytopaenia and the species of malaria is unclear, it is a fact that thrombocytopaenia is universal in malaria-infected patients and should act as a significant indicator of malaria especially *knowlesi* malaria.

Anaemia is also another haematological complication that is usually associated with malaria especially in *P. knowlesi* and *P. falciparum* infections [35,57,78]. The present study also highlighted that anaemia was observed in all species of malaria patients including *P. knowlesi* patients indicating that anaemia is common in human malaria infections. However, malarial anaemia appears to be multifactorial, factors such as genetic disorders (e.g., thalassaemia), haemoglobinopathies, poor dietary intake and socio-economic causes may also contribute to anaemia. It was observed that anaemia is significantly higher in female patients (X^2^ = 5.1; p = 0.025) compared to male patients in the present study. This might also be due to blood loss of female during menstruation. With regard to age, the present study demonstrated that the prevalence of anaemia was significantly higher (X^2^ = 6.30; p = 0.012) in children compared to adults. This is in agreement with other previous studies where malaria-related anaemia is more severe in children rather than in adults [79,80] due to increased demand of iron by the body as growth is rapid in children. In 2002, Praba-Egge and her colleagues [81] carried out experimental infection of non-human primates with *P. knowlesi* and *Plasmodium cynomolgi*. The study reported that anaemia was observed in *P. knowlesi-*infected monkeys after primary and/or repeated infection. Severe anaemia was notable in macaques after repeated infection by *P. knowlesi*[81]. This showed that anaemia was common in both *P. knowlesi*-infected humans or macaques. Malaria-related anaemia is a consequence of hyperparasitaemia and it has been associated with immune-mediated lysis of erythrocytes by schizonts or erythrophagocytosis in the spleen. Findings of the present study showed that both anaemia and thrombocytopaenia are common in malaria-infected patients, including *P. knowlesi* patients.

There was a significant inverse correlation between parasite density with Hb and platelet value for all the species of malaria in the present study. This was in line with other studies where malaria-infected patients tended to have significantly lower Hb levels and platelet counts [9,82]. The trend of increasing parasite density with decreasing platelet count observed in the present study has been previously reported for *P. falciparum*[83,84] but not *P. vivax*[85]. However, detailed study on the relation between parasite density and platelet count for human *P. knowlesi* infection was scarce. This showed that more investigations have to be conducted in order to study the correlation of platelet count with parasite density in malaria infection. Low Hb level due to increased parasite density might be explained by increased haemolysis or decreased rate of erythrocyte production due to the invasion of erythrocytes by malaria parasite [80,86].

White blood cell count (WBC) during malaria are usually characterized as being low to normal [59,87], a decrease in the number of WBC (leukocytes) in the blood is known as leukopaenia whereas elevated WBC refers to leukocytosis. Findings demonstrated that only a small proportion of the malaria patients in the present study suffered leukopaenia and leukocytosis, however, this may be due to the small sample size in the present study. Results showed that the WBC for *P. vivax* malaria patient was lower than *P. falciparum* malaria patient, which was in contrast to previous study where WBC in *P. falciparum* patients were lower than those in *P. vivax*-infected patients and, in turn, lower than those in healthy patients [87]. Overall, the TWBC of patients for all the malaria species detected in the present study were generally within the normal range.

The present study had several limitations. Firstly, haematological profiles such as Hb level, platelet counts and TWBC were only available for 85 patients. The statistical analyses of these parameters were mostly not significant due to the small sample size. Secondly, the baseline demographic data is incomplete and, unfortunately, detailed clinical features of patients could not be retrieved. There were several reasons for the incomplete data in the study. Demographic data and haematological profiles were unable to be retrieved for the samples that were sent from other districts of the division to the clinic. Case notes of all these patients were kept in the respective hospital or clinic therefore the data required were not available. In addition, sometimes, patients with severe malaria were referred to the district hospitals immediately before the full blood profiles were tested in the clinic. There were also some patients with uncomplicated malaria who have refused admission and, therefore, full blood counts were unable to be performed. Thirdly, travel history of patients was not available and therefore the information on where patients acquired malaria infection, especially *P. knowlesi* infection, could not be deduced. Nevertheless, despite all these limitations, this study is one of the few comprehensively analysed studies on the occurrence and epidemiology study of malaria in the eastern districts of Sabah.

## Conclusion

Present study revealed that most of the *P. knowlesi* infection in Sandakan division was misdiagnosed as *P. malariae* by microscopic examination. The wide distribution of human *P. knowlesi* infection indicated that this parasite is generally able to infect humans and there is high potential for *knowlesi* outbreak to occur given that the full knowledge of the dynamics of transmission between human-monkey-mosquito is not adequately understood. This study also observed that there was no clustering of cases of *P. knowlesi* suggesting that transmission may occur in forested areas away from people’s homes. In addition, given that microscopic findings showed that gametocytes were only detected in two *P. knowlesi* patients, indicating that there was less chance of human to human transmission occurring. With regard to haematological analysis, thrombocytopaenia and anaemia was shown to be the most frequent malaria-associated haematological complications. However, larger prospective studies are needed to investigate the relations between parasite species as well as the parasite density to the haematological complications associated with malaria. It is crucial that prospective studies on epidemiological risk factors and transmission dynamics of *P. knowlesi* in these areas are comprehensive in order to accrue accurate epidemiological data, which serves as an imperative foundation in formulating effective malaria control measures that will subsequently contribute to the successful elimination of malaria in Malaysia by 2020. In order to reduce mortality, it is pertinent for malariae suspected samples to be confirmed by PCR. The availability of advanced diagnostic tool PlasmoNex™ enhanced the accuracy and accelerated the speed in the diagnosis of malaria. Given the severity of *knowlesi* malaria and its fatality, confirmed *P. knowlesi* cases should be reported as such by the doctors treating these cases so that the actual disease burden is known.

## Competing interests

The authors declare that they have no competing interests.

## Authors’ contributions

YALL, IV and KHC conceived and designed the experiments. PCL and VN carried out the laboratory diagnosis. XTG, CHC and NJY carried out the molecular genetic studies. XTG, RN and TCT analysed the data. XTG, YALL, TCT and IV drafted the manuscript. KHC, PCL and YALL contributed the reagents/materials/analysis tools. All authors read and approved the final manuscript.
